# Estimating the prevalence of dementia and mild cognitive impairment in Northern Ireland

**DOI:** 10.1002/alz70860_105887

**Published:** 2025-12-23

**Authors:** Nicola A Ward, Calum Marr, Leeanne O'Hara, Charlotte Neville, Michael McAlinden, Claire Potter, David R Weir, Bernadette McGuinness

**Affiliations:** ^1^ Queen's University Belfast, Belfast, Northern Ireland, United Kingdom; ^2^ Northern Ireland Clinical Research Network, Belfast Health and Social Care Trust, Belfast, Northern Ireland, United Kingdom; ^3^ University of Michigan, Ann Arbor, MI, USA

## Abstract

**Background:**

Dementia is a public health challenge, affecting >55 million individuals globally, with an estimated threefold increase by 2050. Cognitive impairment poses significant social and economic burdens, necessitating accurate prevalence estimates and identification of modifiable risk factors. The Harmonised Cognitive Assessment Protocol (HCAP) is an internationally standardised approach using a comprehensive cognitive test battery. It has been adopted across population‐based longitudinal ageing studies to facilitate cross‐country comparisons of cognitive health and dementia prevalence. In Northern Ireland (NI), HCAP was implemented as a sub‐study of the Northern Ireland Cohort for the Longitudinal Study of Ageing (NICOLA). NICOLA‐HCAP aims to provide the first estimates of dementia and mild cognitive impairment (MCI) from a nationally representative sample in NI and assess the role of modifiable risk factors in cognitive decline.

**Method:**

The HCAP subsample consisted of randomly selected community‐dwelling adults aged ≥65 from NICOLA. Participants completed nineteen neurocognitive tests measuring cognitive function across multiple domains and nominated a family member or friend to complete an informant interview on cognitive and functional changes. Research diagnoses of dementia and MCI will be derived using an algorithm based on standard diagnostic criteria, integrating cognitive test performance (≥1.5SD below normative means) and informant‐reported functional decline (e.g., difficulties in daily activities) following Health and Retirement Study measures to ensure comparability of prevalence estimates across the international cohorts. The factor structure of the NICOLA‐HCAP neuropsychological battery will be analysed using confirmatory factor analysis to establish domain‐level cognitive scores.

**Result:**

Almost 50% of eligible NICOLA participants (*n* = 1,037 of 2,077) joined HCAP with 83% (*n* = 862) informant interview completion rate. Participants were 52% female, with higher response rates among younger and more educated individuals. Algorithm development for classifying probable dementia and MCI is ongoing. Planned analyses will examine the impact of modifiable risk factors including sensory impairments, on derived domain‐level cognitive scores including memory, language and executive function.

**Conclusion:**

Results from NICOLA‐HCAP will provide robust prevalence estimates and risk factor insights, essential for investigating the impact of dementia in NI and informing future health and social care policies. Harmonizing NICOLA‐HCAP with other HCAP sub‐studies will support international dementia prevention strategies.

